# Multi-omic applications for understanding and enhancing tropical fruit flavour

**DOI:** 10.1007/s11103-024-01480-7

**Published:** 2024-07-08

**Authors:** Joshua Lomax, Rebecca Ford, Ido Bar

**Affiliations:** Centre for Planetary Health and Food Security, School of Environment and Science, Griffith University, Nathan, QLD 4111 Australia

**Keywords:** Fruit flavour, Sensory analysis, Metabolomics, Transcriptomics, Proteomics, Genomics

## Abstract

Consumer trends towards nutrient-rich foods are contributing to global increasing demand for tropical fruit. However, commercial cultivars in the breeding pipeline that are tailored to meet market demand are at risk of possessing reduced fruit flavour qualities. This stems from recurrent prioritised selection for superior agronomic traits and not fruit flavour, which may in turn reduce consumer satisfaction. There is realisation that fruit quality traits, inclusive of flavour, must be equally selected for; but currently, there are limited tools and resources available to select for fruit flavour traits, particularly in tropical fruit species. Although sugars, acids, and volatile organic compounds are known to define fruit flavour, the specific combinations of these, that result in defined consumer preferences, remain unknown for many tropical fruit species. To define and include fruit flavour preferences in selective breeding, it is vital to determine the metabolites that underpin them. Then, objective quantitative analysis may be implemented instead of solely relying on human sensory panels. This may lead to the development of selective genetic markers through integrated omics approaches that target biosynthetic pathways of flavour active compounds. In this review, we explore progress in the development of tools to be able to strategically define and select for consumer-preferred flavour profiles in the breeding of new cultivars of tropical fruit species.

## Introduction

**Fig. 1:**
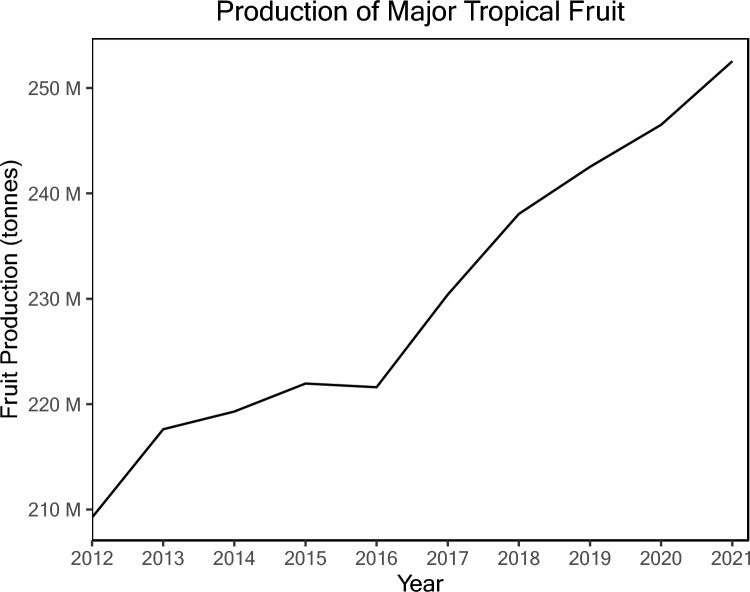
Combined global production of bananas, mango, pineapple, avocado and papaya (FAO 2023)

Tropical fruit species are evergreen, perennial and can produce fruit year-round. The global production value of major tropical fruit crops (bananas, mango, pineapple, avocado and papaya) has steadily increased between 2012 and 2021 by approximately 17%, from ~ 209 Mt to ~ 252 Mt (Fig. 1; FAO 2023) The increase in production is attributed to both growing consumer demand for nutrient-rich “superfoods” and the expanding population, making these fruits lucrative and attractive to farmers in tropical regions (OECD/FAO 2022).

To meet increasing demand and maintain yields, there is a need to continually improve commercial cultivars, particularly due to the threat of tropical plant pathogens and pests. For example, *Fusarium oxysporum* f. sp *cubense* tropical race 4 is a major threat to ‘Cavendish’ banana cultivation (Zakaria 2023). Moreover, the changing climate can significantly impact entire growing regions due to factors such as unfavourable rain distribution, more frequent heat waves and drought. For instance, the production of mango, lychee and pineapple is being affected in sub-tropical regions of Australia due to the increased severity and frequency of extreme weather events (Haque et al. 2020).

Meanwhile, emerging cultivars are generally tailored to meet industry demands, including visual appeal for customers and suitability for long-distance transportation and export. However, fruit flavour has not been included as a priority trait in selective breeding programmes because it is historically difficult to define and select for (Daubeny 1996). The recently developed ‘Goldfinger’ banana is an example of an alternative to the threatened ‘Cavendish’ banana that is an attractive and disease-resistant variety which generates a great yield but has limited improvement for the consumer eating experience (Robertson and Daniells 2023). This has also been reported in tomato (Klee and Tieman 2018), peach (Hancock et al. 2008), blueberry (Gilbert et al. 2014), strawberry (Colquhoun et al. 2012) and papaya (Wills and Widjanarko 1995).

To understand a persons’ preference for tropical fruits, it is important to understand how and why flavour is perceived. An interesting perspective from Castillo (2014) is that flavour is the culmination of smell, taste, mouth sense, sight, sound, emotion, memory, decisions, plasticity, language and consciousness. For this review, we will focus on taste and smell, which are conventionally considered as the predominant contributors to human flavour perception by signalling to the brain from various chemoreceptors on the tongue and in the nasal cavity (Czerny et al. 2008; Kohlmeier 2015). Gustation can be categorised by five taste senses (sweet, bitter, sour, salty and umami) that are detected through three types of taste cells and their corresponding taste receptors, that cluster to form the taste buds on the tongue epithelial tissue (Table 1; Pallante et al. 2021). The biological machinery responsible for olfaction can detect volatile organic compounds (VOCs) that stick to the mucous covering olfactory receptors which are attached to olfactory neurons in the nasal epithelium at the back of the nasal cavity (Castillo 2014). The spectrum of human gustatory and olfactory perception is impacted by genetic variation that impacts each person’s tolerance or preference for different flavours (Robino et al. 2019). Therefore, to characterise fruit flavour attributes across the full spectrum of human flavour perception and how they drive consumer behaviour is an important goal for researchers aiming to advance the flavour quality of tropical fruit.

**Table 1:** Taste cell types and receptors that detect the five conventional taste senses in humans and example of chemicals for each

Taste sense	Taste cell type and receptors	Compound class	Compound example	References
Sweet	II TAS1R2 and TAS1R3	Carbohydrates, proteins, amino acids, peptides, heterocyclic compounds, terpenoids, flavonoids and steroidal compounds	Glucose, fructose, sucrose, D-amino acids, monellin, hesperetin, limonene	Lee and Owyang (2017); Rojas et al. (2022)
Bitter	II TAS2Rs	Alkaloids, phenolic compounds, triterpenes and organosulfur compounds	Caffeine, quinine, catechin, limonin and benzyl isothiocyanate	Drewnowski and Gomez-Carneros (2000); Behrens and Meyerhof (2010); Rojas et al. (2022)
Sour	III OTOP1	Organic acids	Ascorbic acid, citric acid and acetic acid	Behrens and Meyerhof (2010); Calvo and Egan (2015); Rojas et al. (2022)
Salty	I^1^ ENaC	Salts	Sodium chloride and potassium chloride	Calvo and Egan (2015); Pallante et al. (2021)
Meaty or Umami	II TAS1R1 and TAS1R3	Amino acids, short peptides, nucleotide enhancers and organic acids	Glutamic acid, guanosine monophosphate and propionic acid	Calvo and Egan (2015); Pallante et al. (2021); Rojas et al. (2022)

^1^Knowledge gaps exist in the mechanisms for salt sensing; type I taste cells have previously been reported as structural cells in the taste buds (Calvo and Egan 2015; Pallante et al. 2021)

This review explores the concept of consumer preference-driven plant breeding for fruit flavour quality. The expectation being also that stability of preferred flavour types within new cultivars will lead to increased market demand and reduced food waste (Mattsson and Williams 2022; Varese et al. 2023). The review highlights current knowledge on tropical fruit flavour and the methods available to develop tools for selecting and enhancing flavour types. This will inform the development of advanced tropical fruit cultivars that are not only adapted to specific regions and exhibit improved agronomic traits but also deliver superior fruit flavour to consumers.

### Fruit flavour chemistry

The plant metabolites responsible for tropical fruit flavour are compounds that typically also function to deter predators or signal to pollinators and seed dispersers that flowers or fruits are mature (Schaefer et al. 2003). For example, papaya have a high concentration of glucosinolates in the unripe fruit, seeds and fresh leaves (Di Gioia et al. 2020). When these tissues are damaged, the enzyme myrosinase catalyses a reaction that produces the bitter compound benzyl isothiocyanate from the glucosinolate, glucotropaeolin (Rossetto et al. 2008). On the other hand, seed dispersing frugivores are rewarded with essential nutrients from fruits that are detected by the aroma of VOCs which guide animals to consume the fruit (Schaefer et al. 2003; Pech et al. 2008; Nevo et al. 2016). In general, sugars, acids and bitter compounds (e.g. alkaloids) are responsible for the taste of fruits, and the variety of VOCs such as esters, aldehydes, alcohols, ketones, terpenes volatile acids, and volatile sulphur compounds contributes to their aromas (Pérez and Sanz 2008; Klee and Tieman 2018).

### Primary metabolism

Organic compounds, that are initially assimilated by plant tissues, are transformed into primary metabolites (sugars, amino acids, nucleotides, organic acids and fatty acids) to maintain optimum cell function (Maeda 2019). These primary metabolites are important components of human nutrition and contribute to fruit flavour (Sato and Matsui 2012). As for many tropical fruits, the taste of rambutan is attributed to the ratio of sugars (sucrose, glucose and fructose) to acids (such as citric, tartaric, malic, succinic and lactic acids; Deng et al. 2023). In contrast, fatty acids such as palmitic acid, palmitoleic acid, oleic acid and linoleic acid contribute to the unique taste of avocado (Rodríguez et al. 2023).

Sugars and acids are continually synthesised through respiratory metabolic processes including glycolysis, the citric acid cycle and mitochondrial electron transport chain (Fernie et al. 2004). The products of these are involved in many downstream reactions. The building blocks of VOCs include sugars, amino acids, fatty acids and compounds within the methylerythritol 4-phosphate (MEP) and mevalonate (MVA) pathways (Mcgarvey and Croteau’ 1995; Rowan et al. 1999; Liavonchanka and Feussner 2006). However, increased levels of primary metabolites within plant tissues do not directly correlate with increased production of secondary metabolites due to intricate interconnectedness among primary pathways, regulating metabolic functions to maintain plant health and stability (Carrari et al. 2003; Sweetlove et al. 2017).

On the other hand, secondary metabolites are specialised metabolites that allow plants to respond to environmental factors, leading to variations in the types and concentrations of compounds produced. Since they are produced further downstream in the biosynthesis process, they are potentially more amenable to targeted modifications (Martos-Fuentes et al. 2017; Sweetlove et al. 2017). The significance of this difference means that plant breeding initiatives or gene-editing programmes that alter secondary metabolic functions are less likely to introduce negative pleiotropic effects on growth and yield (Sweetlove et al. 2017).

### Secondary metabolism

Many and diverse secondary plant metabolites are responsible for the unique and varied aromas of fruit. These are perceived through VOC production and the development of bitter tastes via production of alkaloids or phenylpropanoids. The flavour of many tropical fruit species is influenced by terpene content, and this is the dominant class of VOC in mango and citrus fruit (Pino and Mesa 2006; Ren et al. 2015). Interestingly, carotenoid compounds, that are part of the same biosynthesis pathway as VOCs, cause the yellow, orange and red colours in tropical fruit but only the downstream products of carotenoids contribute to aroma (Pech et al. 2008; Cazzonelli and Pogson 2010). Carotenoids have a relatively large carbon chain (C_40_) and form VOCs through chemical or enzymatic cleavage into smaller (C_9_, C_10_, C_11_, C_13_ and C_15_) apocarotenoid derivatives, such as β-ionone and β-ionol, which enhance the flavour of carambola fruit (Serra 2015; Jia et al. 2019b).

Generally, the production of volatile aldehydes, alcohols and esters is linked to the degradation of fatty acids that undergo a series of catalytic steps, including through the lipoxygenase pathway (Pech et al. 2008). For example, aldehydes including decanal and (Z)-3-hexenal contribute to the characteristic tone of lime and guava, respectively (Chisholm et al. 2003; Egea et al. 2014). Additionally, the distinct flavours of banana and pineapple are produced by combinations of several alcohol and ester volatiles (Wei et al. 2011; Saha et al. 2018).

In less common cases, other classes of secondary metabolites affect tropical fruit flavour; however, limited information is available regarding their biosynthesis. For example, volatile sulphur compounds, such as diethyl disulfide, contribute to the potent smell of durian and volatile fatty acids, like octanoic acid, can cause papaya to have a pungent aroma (Voon et al. 2007; Adawiyah et al. 2020)..

Information on the biosynthetic pathways for metabolic compounds that underpin the flavour profiles of tropical fruit can be used to focus research into uncovering the governing molecular components. Subsequently, specific enzymes may be targeted to supress or promote the concentration of secondary metabolites that affect fruit flavours. However, first it is imperative to identify the chemical components of fruit flavour that consumers respond to the most.

### Quantifying consumer fruit flavour preferences

Currently, assessment of flavour attributes is performed by trained sensory panel assessors, which is a time-consuming but necessary component to select the future commercial fruit cultivars with the best flavour.

Sensory characterisation, which includes flavour profiling and consumer preference assessments, is the conventional approach used to evaluate fruit taste, aroma, texture and consumer acceptability. Sensory characterisation analysis has been effectively employed to characterise and compare fruit flavour profiles (Egea et al. 2014; Farina et al. 2016; Lasekan and Hussein 2018). Also, to determine the effects of pre- and post- harvest treatments on flavour, and to indicate consumer acceptability of new products or cultivars (Jaeger et al. 2003; Tamby Chik et al. 2011; da Luz et al. 2018), there is a growing body of knowledge on sensory characterisation for many tropical and sub-tropical fruit including: papaya (da Luz et al. 2018), lemon (Serna-Escolano et al. 2022), banana (Arvanitoyannis and Mavromatis 2008), jujube (Galindo et al. 2015), rambutan (Muhamed et al. 2019), loquat (Farina et al. 2016), avocado (Pereira et al. 2014), kiwifruit (Jaeger et al. 2003), guava (Egea et al. 2014), pitaya (dragon fruit; Tamby Chik et al. 2011), durian (Voon et al. 2007) and lychee (Feng et al. 2018). There is less focus on tropical fruit crops that have low market penetration, such as soursop and sapote; however, they have been investigated as food and drink additives to improve consumer acceptability (Pierucci et al. 2019; Khalil et al. 2022). Marques et al. (2022) have conducted an in-depth review on the application of sensory characterisation analysis, focussing on experimental designs that encompass evaluations, lexicon development and statistical analysis.

Generally, sensory surveys are designed to achieve two aims: descriptive analysis and to determine consumer preference or acceptability. Descriptive sensory analysis requires the development of a lexicon including terms that accurately discriminate the decreet flavours perceived upon eating the fruit sample. For example, the predominant characteristics of lychee fruit were determined by seven trained panellists to be cabbage, honey, tropical fruit, garlic/onion, floral, sweetness, astringency, sourness, citrus, green/woody and peach/apricot (Feng et al. 2018), whereas consumer acceptability analysis requires many participants as a subsample of the consumer population because a small survey group will likely result in reduced reproducibility of outcomes (Lande and Thompson 1990; Lawless and Heymann 1999). Sample sizes of approximately 50–100 participants have been implemented in tropical fruit consumer preference studies (Jaeger et al. 2003; Tamby Chik et al. 2011; da Luz et al. 2018). However, larger survey sizes should be included for greater statistical power, such as recent consumer perception research in beverages that include more than 150 participants (Won Kang et al. 2022; Wakihira et al. 2023). The number of participants in larger consumer surveys is likely limited by the cost of hiring participants for the data collection. A novel method that addresses this limitation is the online text highlighting approach, where participants scan a body of text describing the fruit, stopping to highlight descriptors that they like or dislike. For example, Jaeger et al. (2023) surveyed 1140 participants to determine consumer preferences of green and gold kiwi varieties through online recruitment. This approach can reach a significant number of people and filter participants for a balanced representation of the population. However, it requires that people have already tasted the study fruit, which may not be possible for non-commercial cultivars. Interestingly, quick response (QR) codes are becoming ubiquitous on food labels to provide consumers with relevant product information (Fernández-Serrano et al. 2020). Therefore, QR codes may potentially be used to direct consumers to provide feedback on the fruit they have purchased through a citizen science approach. If successful this approach could be used to collect data on many variables including product origin, consumer demographics, environmental data and longitudinal data. However, the efficacy of this approach is untested for fruit flavour surveys and there is evidence that QR code users may be unrepresentative of the actual population (Ozkaya et al. 2015). From a plant breeding perspective, the selection process requires a high-throughput solution which sensory characterisation is not equipped to handle. A promising alternative to consumer surveys involves the use of genetic or metabolomic biomarkers related to specific flavour profiles and consumer perception (Colantonio et al. 2022). Statistical models can be used to develop genetic or chemical assays that target a suite of genes or metabolites relating to flavour-causing biosynthesis pathways, which confer consumer satisfaction or disappointment. These models can reduce the requirement for human subjects in the phenotyping process, leading to increased throughput of samples in fruit flavour research and plant breeding selection. The following sections will explore the development of biomarkers to predict fruit quality in mature stage plants (metabolomics, proteomics, and transcriptomics) and seedling to juvenile stage plants (genomics).

### Development of fruit specific biomarkers for distinct fruit flavour characteristics

#### Metabolomic approaches

Statistical models that predict fruit flavour and consumer acceptance based on metabolite data can increase the tools available to inform post-harvest management and plant breeding selection. For example, fruit quality assays may be implemented to assess for appropriate sugar types and levels. These models require the correlation of quantified flavour-related metabolites with flavour characteristics determined by a trained human sensory panel.

Analytical chemistry methods, particularly chromatography, are employed to measure flavour metabolites in fruit samples. High-performance liquid chromatography is typically used to quantify the concentration of non-volatile metabolites (e.g. sugars, acids, and other secondary metabolites, such as phenolic compounds) and is coupled with a mass spectrometer or refractive index detector to characterise and quantify each metabolite (Suh et al. 2022). Gas chromatography–mass spectrometry paired with a head space solid phase micro extraction step (SPME–GC–MS) is a commonly used approach to target VOCs, which limits the chemical analysis to compounds found in the gas phase being released from the fruit sample (Jouquand et al. 2008; Whye Cheong et al. 2010; Wei et al. 2011; Ren et al. 2015; Zhu et al. 2018; Song et al. 2019; Yang et al. 2019; Brewer et al. 2021). To accurately quantify VOCs in fruit samples, future studies should consider the practices employed by Pico, (Pico et al. 2022), who quantified the VOCs in highbush blueberries (*Vaccinium corymbosum*). Particularly, the inclusion of preservatives, isotope-labelled internal reference standards and spiked sample experiments can provide greater accuracy and identify quantification variability due to interfering compounds within the fruit sample matrix.

Exemplifying the application of LC–MS and SPME–GC–MS paired with sensory characterisation, Sung et al. (2019) correlated the presence and quantities of individual metabolites with sensory characteristics in three mango cultivars. Accordingly, higher liking scores were correlated to high sugar content combined with 1-octanol, (E,Z)-2,6-nonadienal and γ-octalactone; and lower liking scores were correlated to increased amino acid and terpene content. This work can be built upon to develop a predictive model in mango for consumer perceptions. A similar model was developed in papaya by Zhou et al. (2022), who correlated sensory panel data with enzymatic sugar assays and SPME–GC–MS data to determine the role of specific metabolites in five papaya cultivars on consumer flavour perception. From Pearson correlation analysis, glucose, linalool oxide and terpinolene concentrations were negatively correlated with consuming liking scores. Further, a multiple linear regression model (*R*^2^ = 0.991) was developed to predict the consumer liking score of papaya fruit based on the concentration of these three compounds. However, only two yellow fleshed and three red fleshed cultivars were evaluated which may limit the applicability of this model. The accuracy of such models becomes more representative of a species by including a wider variety of genotypes over multiple growing periods. For example, research conducted by Fan et al. (2021) includes 56 cultivars and breeding lines to determine flavour metabolites in strawberry that best explain consumer perceptions. Nonetheless, the model developed for papaya represents the most recent advance in metabolomic selection available in tropical fruit crops. Looking forward, it will be important to consider the use of machine learning models such as XGBoost and gradient-boosting machine. For instance, Colantonio et al. (2022) analysed sensory and chemical datasets for 209 tomato and 244 blueberry samples; it was demonstrated that XGBoost improved the prediction accuracy by an average of 20% and 11% compared to linear regression and Partial Least Squares regression approaches, respectively.

Considering the development of predictive models, like the one for papaya, there is an opportunity to optimise faster detection methodologies compared to LC–MS and GC–MS. For example, qualitative e-nose devices can be calibrated to quickly predict fruit aroma characteristics at a low cost (Salehi 2019; Jia et al. 2019a); and near infrared (NIR) and hyperspectral imaging technologies are promising non-destructive approaches to quantify fruit metabolites (Milczarek et al. 2019). Already, modern imaging technology has been found to be well suited to automation for post-harvest processing to monitor fruit quality such as in the grading of bananas (Mesa and Chiang 2021); and data-rich prediction models are being developed in other crops for fruit quality characteristics. Li et al. (2018) analysed NIR images of 550 cherry fruits to develop a predictive model for soluble solid content and pH values that contribute to fruit quality. Genetic and successive projection algorithms were employed to select the most informative feature bands in the wavelength region of 874–1734 nm; a multiple linear regression model was then produced that incorporated feature bands and the soluble solid content and pH values. Similarly, Hu et al. (2017) correlated feature bands in the visible/NIR wavelength region of 400–100 nm to sugar concentrations in kiwi fruit. Samples treated with 1-methylcyclopropene (to delay the accumulation of soluble sugars), and control samples showed different glucose, fructose and sucrose profiles that were successfully visualised using the developed model.

Once identified, employing metabolomic markers associated with and for the selection of consumer preferences would prove significantly more time- and cost-effective compared to using extensive sensory panel analyses (Colantonio et al. 2022). Although flavour-causing metabolites can explain fruit flavour, understanding the upstream reactions guided by proteins that generate flavour-causing metabolites may bring about novel assays in fruit flavour research.

#### Proteomic approaches

Proteome profiles have been investigated to identify ripening-related proteins in banana (Toledo et al. 2012), guava (Monribot-Villanueva et al. 2022), mango (Andrade et al. 2012) and papaya (Nogueira et al. 2012); the impact of post-harvest treatments on banana (Du et al. 2016) and papaya ripening proteins (Huerta-Ocampo et al. 2012; Der Agopian et al. 2020); allergens and antioxidants in avocado, banana, and mango (Righetti et al. 2015) and proteins that regulate the production of metabolites in mangosteen (Jamil et al. 2021) and papaya (Liu et al. 2018).

In these examples, researchers employ protein extraction (such as trichloroacetic acid (TCA)/acetone, phenol and TCA/acetone/phenol approaches) and digestion (such as trypsin and LysC/trypsin methods) combined with gel- or LC-based separation and MS-based analysis which are typical applications in plant proteomics (Jorrín-Novo et al. 2015; Li and Keller 2023). However, proteome profiling for tropical fruits has previously been considered challenging because of the low protein fraction, high abundance of plant-specific material such as polysaccharides and in some cases solid oil content (e.g. avocado) found in the fruit tissues (Righetti et al. 2014). Potentially, future tropical fruit proteomics research may benefit from combinatorial peptide ligand libraries (CPLL) as an additional step in protein extraction and purification, particularly when targeting low abundance species. This is because CPLL utilises millions of ligands composed of hexapeptides to remove proteins from solution, leaving behind the most abundant proteins (Righetti et al. 2011). A series of three proteomes have been published using CPLL in avocado (Esteve et al. 2012), banana (Esteve et al. 2013) and mango (Fasoli and Righetti 2013); reporting 1012, 1131 and 2855 protein species, respectively.

Another useful approach was employed by Toledo et al. (2012) to determine the protein profiles associated with pre-climacteric and climacteric stages of banana fruit. Two-dimensional fluorescence-difference gel electrophoresis was used to identity differentially expressed protein spots that were excised and prepared for identification using LC–MS/MS. Of the 50 proteins identified, a malate dehydrogenase (MDH, EC1.1.1.37) was detected in the climacteric fruit which catalyses the conversion of oxaloacetate to malate, that is known to influence the sweetness and sourness of banana flesh, along with citrate (Bugaud et al. 2013). Interestingly, ADP-glucose pyrophosphorylase (AGPase, EC2.7.7.27), which plays a role in starch synthesis, was also found to be more active in the ripening banana samples. This provides evidence that starch synthesis and degradation during ripening can happen concomitantly.

Proteome analysis and differential proteome analysis are important to tropical fruit flavour research because it clarifies the mechanisms which control flavour-causing metabolite production in the fruit. There is a benefit to conducting these experiments in tropical fruit because novel enzymes may function in well-known biosynthesis pathways in different species. For example, there is evidence to suggest that enzyme hydroxymethylbutenyl 4-diphosphate synthase (EC 1.17.4.3) is required for carotenoid synthesis in papaya but does not constrain carotenoid synthesis in tomato (Nogueira et al. 2012). Because proteins contribute to fruit flavour throughout biosynthesis pathways, determining catalysts for flavour production may be developed into post-harvest strategies for improving flavour. This has been demonstrated by the application of abscisic acid in kiwifruit to boost the production of aroma compounds that has been supressed by low-temperature storage (Han et al. 2022).

Furthermore, transcriptomics may be able to act as a proxy for protein expression in tropical fruit, although there is evidence to suggest that gene expression levels may not always accurately predict protein expression levels (Liu et al. 2016). This adds value to the data collected by Toledo et al. (2012) for instance, because the proteomics data were able to corroborate previous transcriptomic evidence for gene expression in banana fruit at the same ripening stages. Understanding the role of gene expression in fruit flavour through accurate transcriptome profiling can ultimately provide evidence to the underlying genomic control within the plant.

#### Transcriptomic approaches

Comparative transcriptome analysis is important to understand the molecular components of fruit flavour because plant metabolites are greatly impacted on a transcriptional level (Navarro-Payá et al. 2022). For example, Deng et al. (2018) investigated differentially expressed sequences among purple peel and mature green stage peel of Minhou wild banana (*Musa itinerans*). For this, a de novo transcriptome assembly was implemented due to the low existing mapping rate of the *Musa acuminata* genome (< 20%). Moreover, 65.81% of the previously identified unigenes were unable to be annotated. Despite the paucity of genomic data, of the 3,640 differentially expressed genes (DEGs) identified, 27 were associated with anthocyanin biosynthesis via gene ontology (GO) and kyoto encyclopedia of genes and genomes (KEGG) analyses.

Similarly, Wu et al. (2022) used comparative transcriptome analysis to compare two pitaya cultivars that produced fruit with mild or high grassy aroma. Accordingly, the grassy aroma compounds hexanal and 1-hexanol were significantly associated with the expression of sequences encoding enzymes from the lipoxygenase pathway (*FAD*, *LOX*s, *HPL*s and *ADH*s). The combination of metabolite concentrations and gene expression data, as shown in this example, aided in identifying genes contributing directly to flavour production in the fruit. However, the analysis did not identify sequences related to regulatory elements like transcription factors and microRNAs that influence gene expression, likely due to the lack of an annotated reference genome (Pande 2021).

Analysing these elements requires an understanding of the genes related to the target metabolic pathway and a reference genome to filter likely candidates for analysis. Future research should focus on identifying and targeting these regulatory genes that mediate pitaya aroma. For example, Vallejo-Reyna et al. (2015) applied qRT-PCR to investigate the expression of four ethylene response factor transcription factor genes in papaya; a class of transcription factors that regulate disease resistance pathways. Of these, *CpERF7* was significantly upregulated in response to the bacterial pathogen *Pseudomonas syringae* in one treatment and the plant defence inducer, benzothiadiazole, in another treatment. While targeted RNA studies can elucidate the role of putative transcription factors, future research on gene regulation related to fruit flavour should also explore co-regulatory networks involving transcription factors and microRNAs, which can collectively modify each other’s functions (Pande 2021). Hence, additional information about flavour metabolite pathways is valuable for identifying the likely network of genes and regulatory elements involved.

#### Multi-omics approaches

Incorporating transcriptomics, proteomics and metabolomics can provide in-depth pathway information for tropical fruit flavour. This may be required in some cases because many of the reported metabolic pathways have been characterised for model species such as tomato and may be different in tropical fruit species (Ferrão et al. 2023). Supporting the need for such studies, Jamil et al. (2021) provided evidence in mangosteen, where the enzymes responsible for xanthone biosynthesis (benzophenone synthases) were not functioning as expected from model species. This illustrates the unique metabolic pathways in tropical fruit species, emphasizing the importance of dedicated research in these areas.

When investigating species with limited molecular databases, a multi-omics approach may deliver more accurate results. Yun et al. (2019), for example, characterised metabolic pathways mediating a target trait and identifying related candidate genes by analysing differentially expressed genes, proteins and metabolites in banana fruit. The identification of 5,784 genes, 94 proteins and 133 metabolites differentially expressed or accumulated during banana peel ripening led to the association of genes and pathways with banana peel softening (xyloglucan endotransglucosylase/hydrolase family members). The correlation of metabolites, proteins and gene expression also determined the components of transcriptional regulation (ethylene response factor and basic helix-loop-helix family members), hormone signalling (auxin), aroma synthesis (basic region/leucine zipper motif family and NAC family transcription factors), protein modification (heat shock proteins and ubiquitin-proteasome system) and energy metabolism (anaerobic respiration) related to ripening processes (Yun et al. 2019).

Considering the approach mentioned in the previous section by Wu et al. (2022), a single time point and contrasting samples for the “grassy” aroma trait highlight specific secondary metabolites and the related genes. In contrast, Yun et al. (2019) utilized a different approach, encompassing samples from various ripening stages. This provides more information about genes involved with the fluxes in metabolic pathways that can influence flavour metabolites but do not immediately cause fruit flavour. The benefits of including several time points are further demonstrated by Wang et al. (2022), who reported candidate genes for kiwifruit fruit flavour using a single cultivar. Fruit samples were collected at 12 time points from fruit development to fruit ripening that were each subject to metabolite quantification using GC–MS and mRNA sequencing using the Illumina HiSeq-2000 platform. The dataset was used to construct complex regulatory networks that highlighted structural genes and transcription factors that regulate the biosynthesis of flavour-related metabolites. Experiments that include several time points are necessary to determine the maturation or ripening stages where the peak gene expression, enzyme activity and metabolite synthesis may be observed for the fruit flavour characteristic being researched. This information can be applied to experiments that include the broader germplasm to explore variation across accessions.

Future tropical fruit flavour research should combine the approaches seen in these examples by incorporating sensory characterisation to filter flavour specific pathways in multi-omics approaches; and where possible, target multiple fruit development and ripening stages relevant to the target trait. This is a path towards advanced tools that can improve the speed and efficiency of genetic gain in breeding pipelines which reduce the reliance on sensory characterisation. For fruit flavour however, transcriptomic, proteomic and metabolomic approaches are limited to sampling times when flavour is developing in the fruit. Therefore, to predict fruit flavour in a breeding population at a pre flowering stage, genetic markers would be well suited.

### Development of genomic markers for variations in fruit flavour characteristics

Genomics data may further advance tropical fruit flavour through targeted breeding and genetic selection strategies or gene-editing technology (Mathiazhagan et al. 2021). Genome-wide association studies (GWAS) and quantitative trait loci (QTL) mapping approaches have previously identified genome regions responsible for the regulation of fruit flavour compounds in tomato (Gai et al. 2023; Sapkota et al. 2023), grape (Koyama et al. 2022; Bosman et al. 2023) and apple (Yang et al. 2023).

Genotype by sequencing (GBS) studies of progeny from crossing sweet and unsweet watermelon genotypes have identified a QTL interval (QBRX2-1) strongly associated with sweetness. This was achieved by comparing single nucleotide polymorphisms (SNPs) among the recombinant inbred lines and association with sweetness level (Sandlin et al. 2012; Ren et al. 2014). Ren et al. (2018) subsequently sequenced the QBRX2-1 region in the same population and identified a *Tonoplast Sugar Transporter* (*TST*) gene (*ClTST2*) and a connected sugar-induced transcription factor (*SUSIWM1*) underpinning the sweetness trait. Furthermore, using a GWAS approach comparing QBRX2-1 region sequences and sugar content in 400 watermelon genotypes, *ClTST2* emerged as a key factor in watermelon domestication. Similar *TST* genes were also identified in pineapple (Antony et al. 2008) and fig (Lama et al. 2022).

Long delays in the detection of the genetic variants underpinning a particular target trait may result when using a QTL approach due to the multiple generations required to stabilise recombination. Nevertheless, several QTL studies were conducted in papaya since they are relatively fast growing and early fruiting (Njuguna et al. 2004; Blas et al. 2012; Nantawan et al. 2019). Subsequently, QTL for field measured fruit quality traits were reported, including for sweetness based on total soluble solids. While sweetness plays a significant role in consumer preference (Zhou et al. 2022), future studies should encompass the quantification of sugars, acids and VOCs which can more holistically describe fruit flavour characteristics (Colantonio et al. 2022). Genetic variations linked to specific flavour metabolites will allow greater flexibility to select individuals within a population and hence better outcomes for tropical fruit breeding programmes. Molecular markers representing the gamut of flavour effecting metabolites can then be used to increase the content of desired compounds and/or decrease the content of undesired compounds (Tieman et al. 2017). This approach may be challenging for species or cultivars with limited genomic resources.

Determining the location and function of genetic sequences is dependent on the quality of annotated reference genomes, which appears to be compelling research into new reference genomes. For instance, the transgenic Papaya cultivar, SunUp, was one of the first fruit species to have a reference genome developed (Ming et al. 2008). The genome was recently refined to improve genome completeness, from 72.5 to 98.3% including the annotation of 22,394 protein-coding genes that improved the understanding of sex chromosomes and the effects of domestication, which can improve the success of papaya breeding projects (Yue et al. 2022). This was achieved via the increased accuracy of current sequencing platforms such as PacBio, incidentally also implemented to develop the draft genome of durian (Teh et al. 2017). The availability of crop specific reference genomes is important to identify unique genes that contribute to the production of unique flavour profiles in tropical fruit, such as the sulphur compounds found in durian fruit. New and updated reference genomes for tropical fruit will have a synergistic impact on the accuracy of multi-omic approaches to predict fruit flavour.

There is an opportunity for future research to integrate multi-omic approaches to greatly improve the richness of phenotypic data in projects that aim to develop predictive genetic markers for fruit flavour. However, novel molecular markers need to be validated before their practical use may be realised.

### Molecular tools for the advancement of tropical fruit cultivars

There is continuing progress towards the development and validation of genetic markers linked to fruit flavour characteristics and underlying candidate genes in tropical fruit crops through transcriptomic (Table 2) and genomic approaches (Table 3). The validation of these candidate genes and QTL regions represents a major challenge for researchers in the development of accurate selective genomic trait markers. Interestingly, most of the research into genes responsible for fruit flavour in tropical fruit crops has been conducted using transcriptomics approaches. This may reflect the funding limitations of research in this field, as genomic approaches often demand substantially more time and resources. Also, potentially explaining the lack of validation of uncovered putative candidate genes. Markers associated with gene candidates should be validated on different genetic backgrounds compared to the original population that the candidate genes were discovered. Ideally, genotype to phenotype assessments should be repeated in different environments and over different growing seasons to ensure stability (Sallam et al. 2023). For example, Nashima et al. (2022) identified significant QTL for flesh colour and leaf margin characteristics in pineapple by investigating a biparental population. Using a haplotype resolved reference genome, sequences were identified within each QTL that showed the highest sequence similarity to relevant enzymes previously reported in *Arabidopsis thaliana*. Primers were developed that covered regions of polymorphisms that were linked to each trait that were subsequently tested on 41 pineapple accessions. The genotype characterised by the genetic markers was in complete agreement with the flesh colour and leaf margin phenotype of all accessions, making a strong case that these markers were stable, accurate and can be implemented into pineapple breeding programmes.

**Table 2:** Transcriptome research to identify genes related to fruit flavour in tropical fruit crops

Crop	Description	Significant genes	Expression analysis platform	References
Avocado	Aroma-related genes effected by ethylene	*LOX*	qRT-PCR	García-Rojas et al. (2016)
Banana	Expression analysis of genes relating to volatile biosynthesis	*BCAT*, *LOX*, *HPL*, *PDC*, *ADH*, *AAT*	qRT-PCR	Yang et al. (2011)
	Genes related to aroma formation	*TGL*, *CAT*	BGISEQ-500	Dou et al. (2022)
	Sugar metabolism-related genes	*CwINV*	qPCR	Fils-Lycaon et al. (2011)
Carambola	Expression analysis of sugar transporter gene family	*SWEET*	qRT-PCR	Lin et al. (2021)
Custard apple	Genes related to cell wall polysaccharide degradation	*EG*, *β-Glc*, *β-xyl*, *GATU*, *PME*, *Pel*,* PG*	Illumina HiSeq X Ten	Wang et al. (2023)
	Sugar accumulation-related genes	*SPS*, *ISA*, *AMY*, *BAM*, *AGL*	Illumina HiSeqTM4000	Fang et al. (2020a)
Jackfruit	Sugar metabolism-related genes	*AINV*, *SUSY*, *AGPase*, *SS*, *SBE*, *GAUT*, *pectinesterase*, *CYC-ß*, *PSY*	Illumina HiSeq 2500	Hu et al. (2016)
Longan	Expression analysis of sugar transporter gene family	*INT*, *pGlcT*, *PLT*, *STP*, *VGT*	qRT-PCR	Fang et al. (2020b)
Lychee	Expression analysis of sucrose synthase genes	*SUSY*	qRT-PCR	Wang et al. (2021)
Mango	Sugar accumulation-related genes	*NAC*, *MYB*, *SWEET*, *SUC*, *SPP*, *INV*, *SUSY*, *HK*, *FK*	Illumina HiSeq 2500	Li et al. (2020)
	Gene networks regulating fruit quality and volatile compounds	*CS*, *ACO*, *IDH3*, *DLST*, *LSC2*, *SDH2*, *fumC*, *MDH2*, *DLAT*, *PDHA*, *PDHB*, *BCDH*, *mmuM*, *metE*, *MGL*, *MetK*, *ACS*, *ACO1*, *SAHH*	Illumina NextSeq	Li et al. (2023)
	Genes related to aroma formation	*E5.5.1.13*, *KAO*, *K15095*,	Illumina HiSeq 2500	Xin et al. (2021)
Pineapple	Sugar transporter-related genes	*SWEET*, *ZIFL*, *STP*, *SUT*, *pGLCT*, *SUT*, *STP*,	qRT-PCR	Fakher et al. (2022)
	Expression analysis of sugar transporter gene family	*SWEET*	qRT-PCR	Lin et al. (2022)
	Sugar transporter-related genes	*SWEET*	Illumina HiSeq 2500	Guo et al. (2018)
Pitaya (dragon fruit)	Genes associated with sugar and organic acid metabolism	*INH*, *INV*, *HK*, *PFK*, *SUT*, *SWEET*, *PK*, *PDH*, *CS*, *ACO*, *NADP-IDH*, *GAD*, *MDH*, *H* + *-ATPase*, *ALMT*, *NaDC*	Illumina HiSeq 2000	Gao et al. (2022)
	Genes associated with sugar and organic acid metabolism	*APX*, *MDAR*, *PMI*, *AMY*, *BAM*, *PHS*, *APGS*, *SPS*, *Ivr*, *SUSY*, *HXK*, *FRK*, *PGI*	Illumina Novaseq	Xie et al. (2022)
	Betalain biosynthesis related to flesh colour	*CYP76AD4*, *DODA*,* NAC*	Single-Molecule Real-Time sequencing and Illumina RNA-Seq	Wu et al. (2020)
Papaya	Sugar-synthesis-related genes	*SPS*, *CWINV*, *AVIN*	qPCR	Nantawan et al. (2018)
	Genes related to aroma formation	*TPS10*, *CYP76C1*, *CYP71B31*, *AAT*, *ACX*	qPCR	Liu et al. (2019)
	Genes related to sugars and VOC metabolism	*GES*, *BEBT*, *STP*, *PFP*, *transglycosidase*, *BoGH3B-like*, *GPT*	NanoString Elements Tagset	Zhou et al. (2022)
Passionfruit	Expression analysis of lipoxygenase gene family	*LOX*	qRT-PCR	Huang et al. (2022)
	Genes related to aroma formation during ripening	AAT, LOX, HPL and PAL	HiSeqTM 2500/Illumina HiSeq X Ten	Li et al. (2021)

**Table 3:** Association mapping research to identify markers of fruit flavour characteristics in tropical fruit crops

Crop	Description	Genotyping platform	References
Banana	QTLs related to organoleptic traits	Illumina Hiseq 3000	Biabiany et al. (2022)
Guava	QTLs for vegetative and reproductive characters	AFLP markers	Rodríguez et al. (2007)
	QTLs for fruit quality traits (QTL mapping)	SSR/EST-InDels assay and EST-SNP/KASP assay	Maan et al. (2023)
Longan	QTLs for soluble solids and seed weight	Illumina NovaSeq 6000	Wang et al. (2022)
Mango	QTLs for fruit colour and firmness	Affymetrix mango genome 80 K SNP genotyping chip array	Srivastav et al. (2023)
Papaya	QTLs related to sweetness and other fruit quality traits	Illumina HiSeq 2500	Nantawan et al. (2019)

While the application of gene-editing approaches is limited due to the cost of development, intellectual property issues, public concern and regulatory burdens, it can be used to accurately edit candidate genes for functional validation (Langner et al. 2018). Genes related to disease resistance, beta-carotene enrichment and extended fruit shelf-life have been functionally proven using gene-editing methods in banana, papaya, citrus and melon (Nizan et al. 2020; Mathiazhagan et al. 2021). This has also been employed in a multiplex manner to simultaneously delete multiple gene functions in tomato and rice (Langner et al. 2018). Since many traits including flavour are likely governed by multiple genes, the ability to knock out panels of genes using this approach is powerful. However, researchers must consider that loss-of-function phenotypes related to edited alleles can exaggerate the effect of target genes compared to natural variations that may confer subtle changes (Gudmunds et al. 2022).

Gene knock-down is also possible using an RNAi approach to investigate the function of candidate genes. Fragments of the candidate gene are introduced into the plant and spliced into short RNAs to induce gene silencing through the endogenous RNA interference pathway (Kumar et al. 2018). The target sequence can be delivered to the plant by a virus vector using virus-induced gene silencing (VIGS) or direct methods including transgenic approaches (host-induced gene silencing; Qi et al. 2019). Tzean et al. (2019) successfully demonstrated this phenomenon in banana plants using a banana-infecting cucumber mosaic virus isolate (CMV20) as a vector to silence *glutamate 1-semialdehyde aminotransferase* (GSA) and *phytoene desaturase* (PDS) transcripts, resulting in chlorosis and photobleaching of leaf tissues, respectively. They demonstrated that CMV20 VIGS injected into the pseudostem-rhizome of each plant reached an infection rate of 95%. Furthermore, infected plants exhibited reduced transcription of 10% and 18% for GSA and PDS, respectively, in comparison to control plants. Conducting preliminary optimisation studies using VIGS is crucial because the duration of gene silencing can be unpredictable due to the transient nature of the approach. This unpredictability makes it challenging to accurately measure the effects on the phenotype, which can also vary in strength between tissues. Future experiments may refer to Dommes et al. (2019) who describe important steps in experimental planning for VIGS applications.

Future marker-assisted selection in tropical fruit crops may benefit from machine learning applications for genetic modelling (Hayes et al. 2023). For instance, the R package ‘genomicSimulation’ can analyse molecular markers within a germplasm population to optimize parental crosses for genetic gains (Villiers et al. 2022). By incorporating sensory data within the pipeline for marker development, it becomes feasible to integrate fruit flavour characteristics into the balanced breeding of new tropical fruit cultivars.

## Conclusion

In conclusion, tropical fruit species represent an important economic resource and contribute to a healthy human diet and continual breeding for commercial cultivars is required to address changes in market demands, climate conditions and pest and disease pressures. Multi-omic approaches may be used to develop biomarkers associated with consumer-preferred fruit flavour in breeding programmes. Through the characterisation of diverse flavour profiles within a crop species, preferred and novel flavours can be linked to specific metabolites and their corresponding metabolic pathways, leading to the identification of flavour correlated genes. With the continual advances in genome sequencing and editing, it is likely that functional genetic markers will become a viable addition to tropical fruit breeding programmes for polygenic traits such as fruit flavour. The incorporation of consumer preferences in the development of selection tools for new tropical fruit cultivars will ensure that optimal fruit flavour is retained or improved throughout the breeding process.

## Data Availability

Enquiries about data availability should be directed to the authors.
